# The risks of hematological toxicities of nivolumab in cancer patients: A PRISMA-compliant meta-analysis

**DOI:** 10.1097/MD.0000000000032393

**Published:** 2022-12-30

**Authors:** Zuolin Shi, Xiyu Liu, Mengjia Chen, Na Zhang, Hongna Guan, Dongyang Ye

**Affiliations:** a Department of Neurosurgery, General Hospital of Northern Theater Command, Shenyang, PR China; b Department of Neurology, No.926 Hospital, Joint Logistics Support Force of PLA, Yunnan, PR China; c Department of Officers, General Hospital of Northern Theater Command, Shenyang, PR China; d Department of General Surgery, Shengjing Hospital of China Medical University, Shenyang, PR China.

**Keywords:** cancer, hematological toxicity, nivolumab, programmed cell death-1 (PD-1) inhibitor

## Abstract

Nivolumab is the human programmed cell death-1 (PD-1)-blocking antibody showing significant effect in many refractory cancers. However, little is known about its risks of hematological toxicities, rare but clinically serious and potentially life-threatening adverse events. We want to explore whether nivolumab can increase the risks of hematological toxicities compared with other immunotherapy or chemotherapy drugs.

**Method:**

The databases of PubMed, Embase, Web of science, and CNKI were searched. We used the medical subject heading terms “Nivolumab” plus keyword “Nivolumab” to search studies published from August 1990 to October 2021. For the included articles, we calculated the relative risks and the corresponding 95% confidence intervals (CIs) for the risks of anemia, neutropenia, and leukopenia in patients treated with nivolumab versus control drugs.

**Results:**

Five original articles on the nivolumab trials were identified with 2399 patients enrolled in this meta-analysis. The relative risks of anemia, neutropenia, and leukopenia were 0.343 (95% CI: 0.177–0.663; *P* = .001), 0.020 (95% CI: 0.008–0.053; *P* = .000), and 0.054 (95% CI: 0.015–0.191; *P* = .000), respectively.

**Conclusion:**

The PD-1 inhibitor-nivolumab did not increase the risk of anemia, neutropenia and leukopenia. It may enhance awareness about lower risks of hematological toxicities when choosing nivolumab as PD-1 inhibitor among clinicians.

## 1. Introduction

Immunotherapy is a new treatment strategy for cancer patients in recent years. Some well-known suitable targets for cancer immunotherapy include cytotoxic T lymphocyte antigen-4 and programmed cell death-1 (PD-1).^[1,2]^ PD-1 is one of the significant immunosuppressive molecules and the most well-established co-inhibitory regulators suppressing proliferations and cytokine’s productions of T-cells. As a member of the CD 28 protein superfamily, it can limit T-cells’ cytokine secretion, function and proliferation, thus having an important significance for cancer prevention and therapy, anti-inflammation, anti-autoimmune diseases and organ transplantation.^[3]^ Recently, Lim et al found that PD-1 expressions on dendritic-cells could significantly suppress CD8^+^ T-cells’ functions and antitumor-immunities, which provided a more comprehensive understanding of PD-1’s roles in immune-regulations.^[4]^ Clinical trials were also being carried out to estimate the benefits of nivolumab in combination with other drugs. The most commonly used and standard therapeutic options with nivolumab have been developed for patients with melanoma, non-small-cell lung cancer, renal cell carcinoma urothelial carcinoma, and head and neck squamous cell carcinoma.^[5–7]^ Other advanced cancers include gastric cancer, gastro-esophageal cancer, Hodgkin lymphoma, ovarian cancer, and esophageal cancer.^[8,9]^ Rajan et al reviewed relevant clinical trials of nivolumab’s effects for advanced cancer patients and found that although nivolumab could be well-tolerated, its T-cell response hyperactivation led to normal tissue damage or organ system failure, what’s more, its hematologic toxicity was potentially life-threatening.^[10]^ However, large heterogeneity between data sources in the incidence of hematological adverse-events among clinical trials like anemia, neutropenia, and leukopenia, etc was noted.^[11–14]^ There were still no comprehensive reviews or analyses to pool these data. That’s why we performed this meta-analysis of previously published randomized clinical studies.

## 2. Methods

### 2.1. Search strategy and study selection

The databases of PubMed, Embase, Web of science, and CNKI were searched. We used the medical subject heading terms “Nivolumab” plus keyword “Nivolumab” to search studies published from August 1990 to October 2021. We only include randomized controlled trials. No language limitations were set. If the 2 independent investigators had disagreements, they would discuss together with the third investigator. Table 1 clearly showed the inclusion criteria and the exclusion criteria for this meta-analysis. In order to avoid missing potential studies, we also scanned the abstracts of the references in each included article.

**Table 1:** Inclusion criteria for study selection in this meta-analysis.

Number	Inclusion criteria
1	Randomized phase II and III studies in patients with solid tumors
2	The outcome of the study includes hematological toxicities like anemia, neutropenia, and leukopenia
3	Participants received treatment with nivolumab
4	The relative risk (RR) with a 95% confidence interval (CI) of the risk of selected hematological toxicities associated with nivolumab could be obtained from articles directly or calculated based on the figures or tables given in articles, or through contacting the authors
5	For the duplicate articles, only the most complete or the most newly published one was included
Number	Exclusion criteria
1	Phase I trials were excluded
2	The treatments in experimental groups should not include other drugs

CI = confidence interval.

### 2.2. Data extraction

The data were extracted by 1 primary investigator (ZS) and then reviewed independently by 2 secondary investigators (XL and NZ). Firstly, we made a table with the first line showing which kind of data should be extracted. The extracted data included author names, publication years, which phase the clinical trial belonged to, the patient numbers in each group, the intervening measures and so on. We mainly focused on the side effects of the hematologic system including anemia, neutropenia, and leukopenia. We adopted the Jadad quality assessment as the method to evaluate the literatures’ quality.

### 2.3. Statistical analysis

Review Manager 5.3 (The Cochrane Collaboration, Oxford, UK) and STATA 13.0 (StataCorp LP, College Station, TX) were used for statistical analysis. The overall scheme determined whether the data from different studies had heterogeneity; if yes, we used Laird and DerSimonian random-effects model; if no we used the fixed-effects model; performed a sensitivity analysis to determine whether the pooled results were stable; and performed contour-enhanced funnel plots to determine whether the included studies had publication bias. We firstly used Labbe plots, *I*^2^ tests and Cochran *Q* test to determine whether the data from all the studies had heterogeneity. The Cochran *Q* test was able to test differences among >3 matched sets of proportions or frequencies. *P* < .05 was regarded as statistical significances. The sensitivity analysis method was the 1-at-a-time method: omitting each study at each time and repeating the whole process of the results’ pooling to see if the final results were robust (Table 2).^[15]^ As only 5 studies were included, the meta-regression analysis was not necessary to conduct.

**Table 2:** The statistical methods used in this meta-analysis.

Statistic means	Goals and usages
Labbe plot	To evaluate heterogeneity between the included studies
Cochran *Q* test	To evaluate heterogeneity between the included studies
*I*^2^ index test	To evaluate heterogeneity between the included studies
Sensitivity analysis	To examine the stability of the pooled results
Contour-enhanced funnel plot	Publication bias test

## 3. Results

### 3.1. Search results and characteristics of the studies

**Figure 1.**
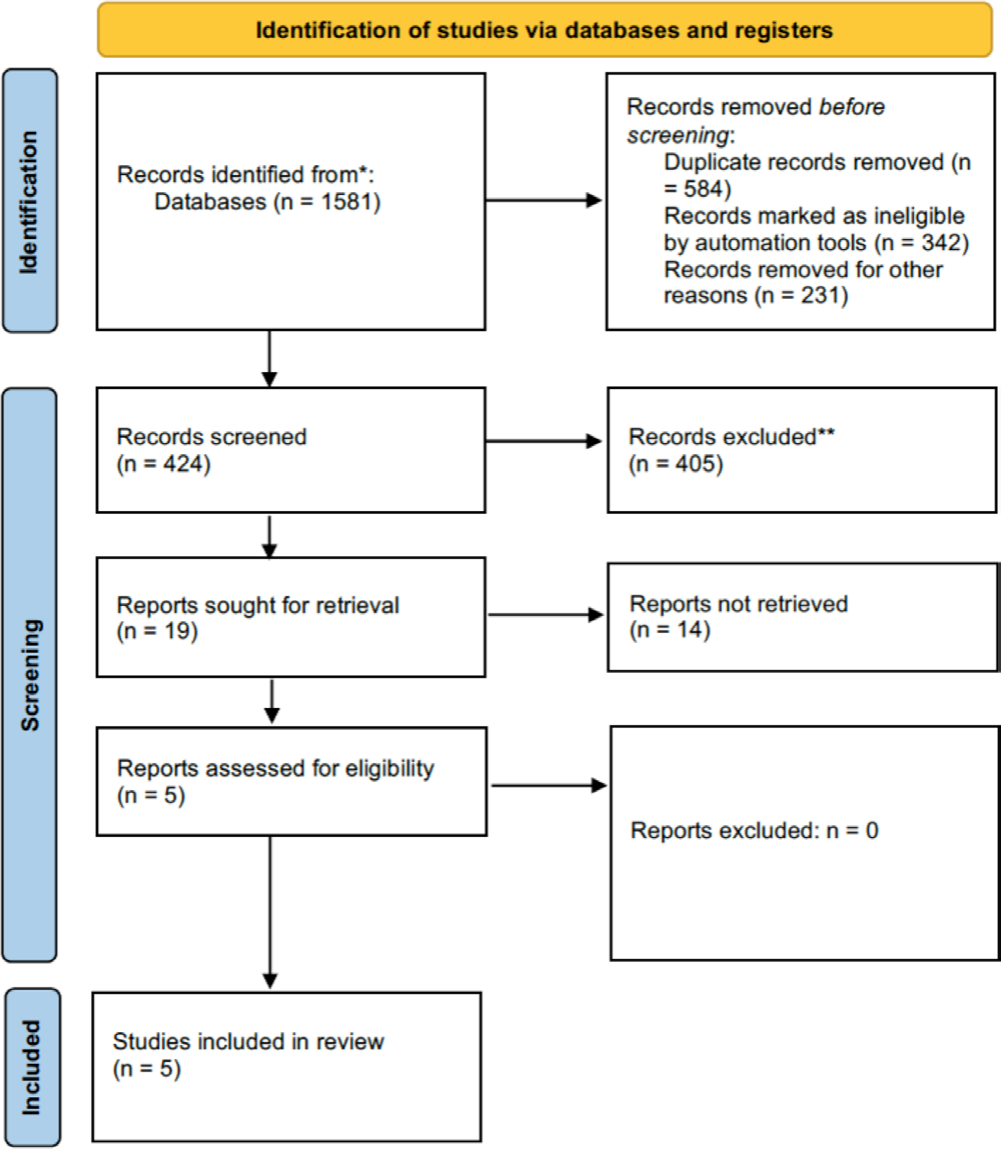
Literature search and selection of articles.

The entire flow of the literature-search process was shown in Figure 1. We eventually enrolled 5 randomized phase III trial studies including 2399 subjects,^[7,11–14]^ which were all written in English (4 were finished in the US^[7,11,12,14]^ and 1 in France^[13]^). The basic information including the treatment arms and interventions for each group was presented in Table 3.

**Table 3:** Baseline characteristics of included studies comparing nivolumab to chemotherapy drugs.

Study	Yr	Country	Study type	Treatment arms	Indication	Anemia	Neutropenia	Leukopenia
Robert	2014	France	Phase III	Arm A: Nivolumab 3 mg/kg of body weight every 2 weeks (206 pts) Arm B: dacarbazine (205 pts)	Stage III or IV unresectable melanoma without a BRAF mutation	0 (0%) versus 23 (11.2%)	9 (4.4%) versus 1 (0.5%)	1 (0.5%) versus 7 (3.4%)
Weber	2015	USA	Phase III	Arm A: Nivolumab 3 mg/kg of body weight every 2 wk (268 pts) Arm B: Investigator choice chemotherapy (102 pts)	Patients with advanced melanoma who progressed after anti-CTLA-4 treatment	12 (4.5%) versus 23 (22.5%)	0 (0%) versus 19 (18.6%)	–
Borghaei	2015	USA	Phase III	Arm A: Nivolumab 3 mg/kg of body weight every 2 weeks (292 pts) Arm B: docetaxel (290 pts)	Advanced non-squamous cell NSCLC	34 (12%) versus 68 (25%)	2 (1%) versus 87 (32%)	0 (0%) versus 22 (8%)
Brahmer	2015	USA	Phase III	Arm A: Nivolumab 3 mg/kg of body weight every 2 wk (131 pts) Arm B: docetaxel (129 pts)	Advanced squamous cell NSCLC	2 (2%) versus 28 (22%)	1 (1%) versus 42 (33%)	1 (1) versus 8 (6%)
Motzer	2015	USA	Phase III	821 patients were randomly assigned (in a 1:1 ratio) to receive 3 mg of nivolumab/kg of body weight intravenously every 2 wk or a 10 mg everolimus tablet orally once daily	Advanced clear-cell renal cell carcinoma for which they had received previous treatment with 1 or 2 regimens of antiangiogenic therapy	32 (8%) versus 94 (24%)	–	–

BRAF = B-Raf, CTLA-4 = cytotoxic T lymphocyte antigen-4, NSCLC = non-small cell lung cancer.

### 3.2. Quality analysis

The Jadad scale (Table 4) items included randomization, blinding and an account of all patients in addition to the overall score. The overall score was 5 in 1 study^[13]^ and 3 in the other 4 studies.^[7,11,12,14]^

**Table 4:** Jadad quality assessment of the included studies.

Study	Yr	Country	Study type	Randomization	Blinding	An account of all patients	Overall score
Robert	2014	France	Phase III	2	2	1	5
Weber	2015	USA	Phase III	2	0	1	3
Borghaei	2015	USA	Phase III	2	0	1	3
Brahmer	2015	USA	Phase III	2	0	1	3
Motzer	2015	USA	Phase III	2	0	1	3

### 3.3. Hematological toxicity

**Figure 2.**
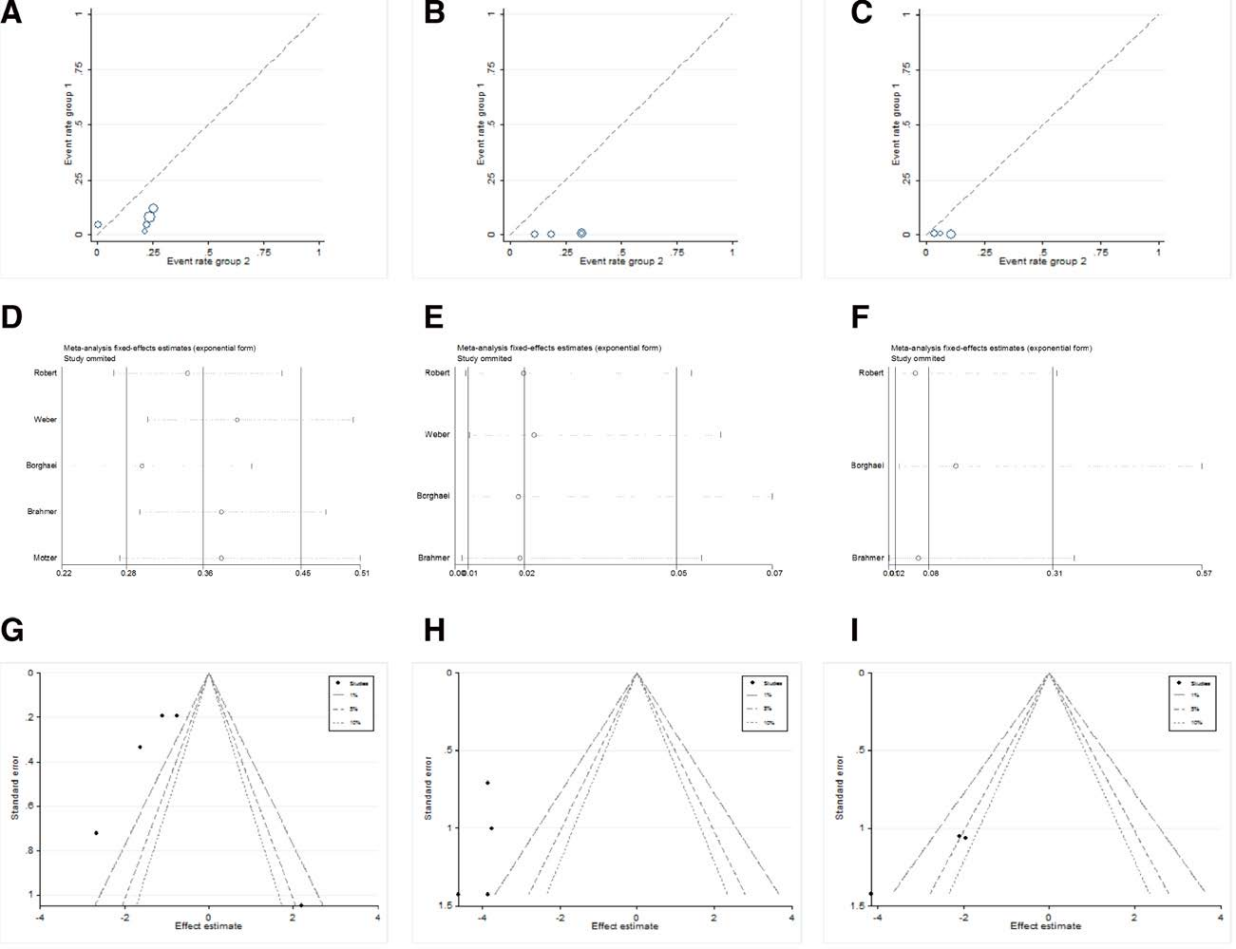
Labbe plots, sensitivity analysis plots and contour-enhanced funnel plots of the included studies focusing on the risk of selected hematological toxicity associated with the PD-1 inhibitor nivolumab Labbe plots concerned (A) anemia, (B) neutropenia, and (C) leukopenia, respectively. Sensitivity analysis concerned (D) anemia, (E) neutropenia, and (F) leukopenia. Contour-enhanced funnel plots concerned (G) anemia, (H) neutropenia, and (I) leukopenia. PD-1 = programmed cell death-1.

**Figure 3.**
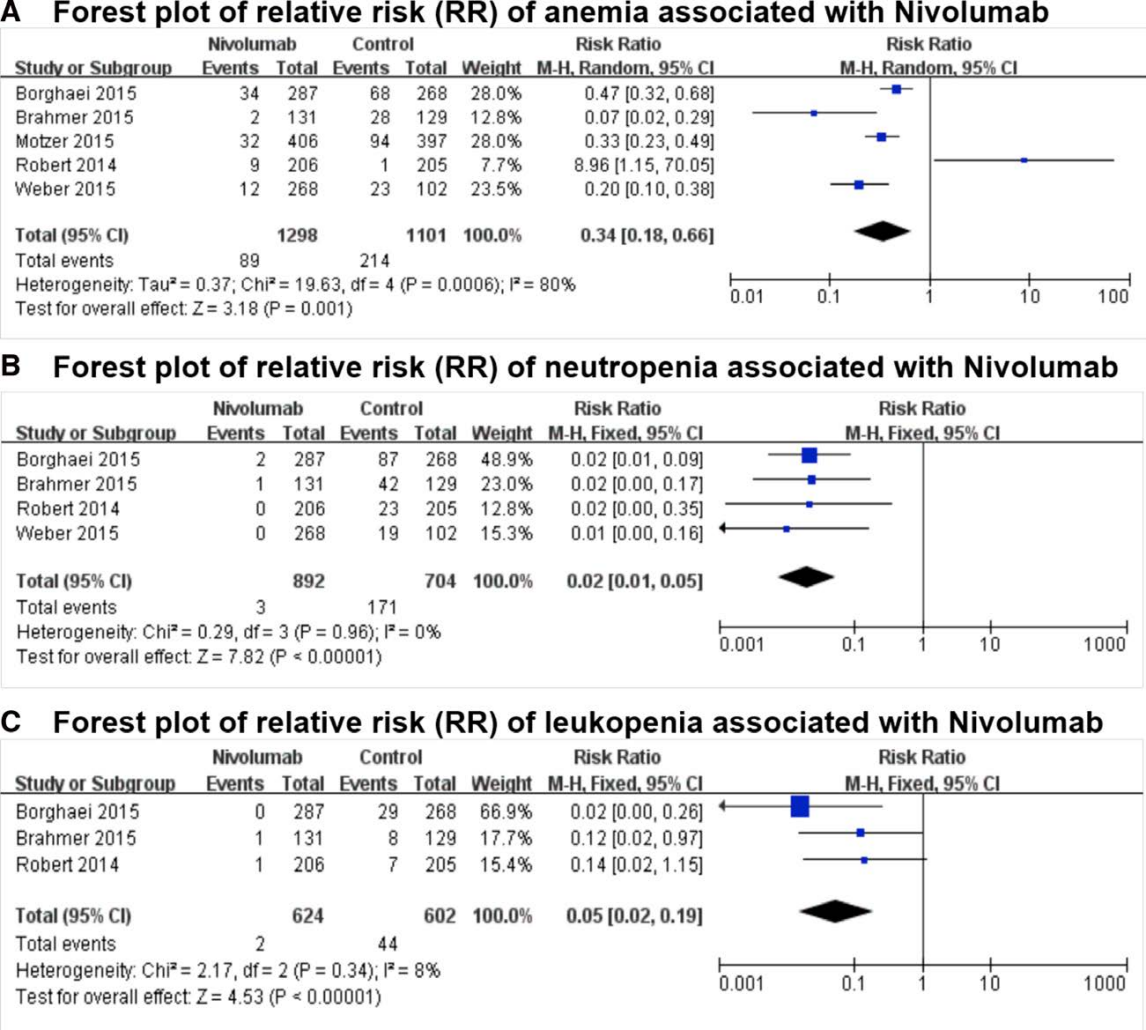
Forest plots (individual and pooled effects with 95% CI) regarding the risks of selected hematological toxicity of (A, random effect model) anemia, (B, fixed effect model) neutropenia, and (C, fixed effect model) leukopenia associated with nivolumab versus controls. CI = confidence interval.

Anemia was reported in all 5 studies; neutropenia was reported in 4 studies; and leukopenia was reported in 3 studies. The Labbe figure (Fig. 2A) suggested the heterogeneity among these researches (*Q* = 19.63, degree of freedom [df] = 4, *I*^2^ = 79.6%, *P* = .001), thus the random-effect model was used. As revealed in Figure 3A, the forest plot demonstrated that nivolumab was associated with a decreased risk of anemia (relative risk [RR] = 0.343, 95% confidence interval [CI]: 0.177–0.663; *P* = .001).

We tested the correlation of the risk of neutropenia, leukopenia with nivolumab versus with the control like dacarbazine, docetaxel, or everolimus on the according effect models (neutropenia: *Q* = 0.29, df = 3, *I*^2^ = 0.0%, *P* = .963, fixed-effect model, Fig. 2B; leukopenia: *Q* = 2.17, df = 2, *I*^2^ = 7.9%, *P* = .337, fixed-effect model, Fig. 2C). The results also suggested a lower incidence of hematotoxic adverse events in patients treated with nivolumab versus the control in issues of neutropenia (RR = 0.020, 95% CI: 0.008–0.053; *P* = .000; Fig. 3B), and leukopenia (RR = 0.054, 95% CI: 0.015–0.191; *P* = .000; Fig. 3C).

### 3.4. Sensitivity analysis and publication bias

From Figure 2D–F, we can see the pooled results would not be influenced by removing any one study from all the included studies. The contour-enhanced funnel plots showed that the missing areas for the included studies were in the right-hand side of the figure, revealing no publication bias (Fig. 2G–I).

## 4. Discussion

This meta-analysis regarding the evaluation of hematological toxicities (anemia, neutropenia, and leukopenia) in cancer patients treated by nivolumab demonstrated that nivolumab was associated with a decreased risk of hematological toxicities compared with other similar drugs.

Normally, T cells could kill cancer cells and be controlled by inhibitory-checkpoints to avoid attacking normal tissues. So, downregulating these kinds of checkpoints could activate T cells to have stronger anti-cancer capacities.^[16]^ PD-1 had recently been an important and hopeful checkpoint. PD-1 inhibitor-nivolumab played a significant role in anticarcinogenic actions, which had been proved by several phase II to III clinical trials.^[17–20]^ The hematological adverse effects were significant reasons for treatment interruption or discontinuation. As so far, there were still not enough effective ways to predict patients with high risks of hematological adverse effects, so close clinical monitoring of laboratory or clinical indexes were important. Hematological toxicities had been also reported in a number of other targeted anticancer therapeutics and had been linked to noncompliance with many of them.^[7,11–14]^ In this article, we analyzed the correlation of the risk of anemia, neutropenia, and leukopenia with nivolumab versus the control drugs like dacarbazine, docetaxel, or everolimus systematically. We found that in comparison with the controls, nivolumab did not increase the risks of hematological toxicities of anemia, neutropenia, and leukopenia. Therefore, although clinicians using nivolumab should be attentive of its side effects, in term of hematological system side effects, nivolumab was not more dangerous than others.

There were some limitations in this meta-analysis. First, it was performed at the study level rather than analyzing the potential variables at the patient level. Second, different doses, frequencies of nivolumab, or different control drugs, or different types of cancers may be sources of heterogeneity. Third, contour-enhanced funnel plots were used to evaluate the publication biases of this meta-analysis. One black point represented a study. If most points fell into the region of lower statistical significance (the left area of the plot), then we could deem it as an evidence of no publication bias. Otherwise, the publication bias should be suspected. In the present meta-analysis, although the funnel plot indicated no publication bias, probably underreporting of small, negative, or non-significant data in the published literature was not included.

## 5. Conclusions

Summarily, PD-1 inhibitor-nivolumab as a new treatment strategy for cancer patients did not increase the risks of hematological toxicities including anemia, neutropenia, and leukopenia. It may enhance awareness about lower risks of hematological toxicities when choosing nivolumab as PD-1 inhibitor among clinicians.

## Author contributions

Zuolin Shi, Xiyu Liu, Mengjia Chen and Na Zhang performed writing the manuscript and analyzed and interpreted the data. Hongna Guan and Dongyang Ye were major contributors in study design.

**Conceptualization:** Zuolin Shi.

**Data curation:** Zuolin Shi, Xiyu Liu, Na Zhang.

**Formal analysis:** Zuolin Shi, Xiyu Liu, Mengjia Chen.

**Funding acquisition:** Hongna Guan, Dongyang Ye.

**Investigation:** Zuolin Shi, Xiyu Liu, Hongna Guan.

**Methodology:** Zuolin Shi, Na Zhang, Hongna Guan.

**Project administration:** Xiyu Liu, Hongna Guan.

**Resources:** Zuolin Shi, Na Zhang.

**Software:** Zuolin Shi, Na Zhang, Dongyang Ye.

**Supervision:** Hongna Guan, Dongyang Ye.

**Validation:** Hongna Guan.

**Visualization:** Xiyu Liu, Mengjia Chen, Na Zhang, Hongna Guan, Dongyang Ye.

**Writing – original draft:** Zuolin Shi.

**Writing – review & editing:** Zuolin Shi, Xiyu Liu, Mengjia Chen, Na Zhang, Hongna Guan, Dongyang Ye.
